# Acupuncture for Functional Dyspepsia: What Strength Does It Have? A Systematic Review and Meta-Analysis of Randomized Controlled Trials

**DOI:** 10.1155/2016/3862916

**Published:** 2016-12-29

**Authors:** Bo Pang, Tao Jiang, Yuan-Hao Du, Jing Li, Bo Li, Ya-Cai Hu, Qiu-Han Cai

**Affiliations:** ^1^Department of Acupuncture and Moxibustion, First Teaching Hospital of Tianjin University of Traditional Chinese Medicine, Tianjin, China; ^2^Key Laboratory of Acupuncture of Tianjin, First Teaching Hospital of Tianjin University of Traditional Chinese Medicine, Tianjin, China

## Abstract

*Background*. Although the effectiveness of acupuncture therapy on functional dyspepsia (FD) has been systematically reviewed, the available reports are still contradictive and no robust evidence has been provided to date.* Objective*. To assess the current evidence of high quality on the effects of acupuncture for patients with FD.* Methods*. A comprehensive literature database search was conducted to identify randomized controlled trials (RCTs) comparing acupuncture therapies (including manual acupuncture and electroacupuncture) to sham acupuncture and medication use. A meta-analysis was performed following a strict methodology.* Results*. 16 RCTs involving 1436 participants were included. The majority of the trials were determined to be of low quality. Positive results were found for acupuncture in improving the Nepean Dyspepsia Index (NDI) and scores of the MOS 36-Item Short-Form Health Survey (SF-36), as well as in alleviating relevant symptoms (especially postprandial fullness and early satiation) of FD patients.* Conclusion*. Based on current available evidence, acupuncture therapy achieves statistically significant effect for FD in comparison with sham acupuncture and is superior to medication (prokinetic agents) in improving the symptoms and quality of life of FD patients. Nonetheless, despite stringent methodological analyses, the conclusion of our review still needs to be strengthened by additional RCTs of higher quality.

## 1. Introduction

“Bad digestion,” the first description of dyspepsia more than 2000 years ago, has been commonly bothering people worldwide [1–3]. As the most common cause of the symptom constellation of dyspepsia, functional disorder of upper gastrointestinal (GI) tract has been investigated for decades [1, 4]. However, despite the high prevalence of functional dyspepsia (FD), the etiology and pathophysiology of this disorder remains poorly understood. According to surveys, the prevalence of FD has been noted to vary between 11% and 29.2% [5, 6]. Although the current endorsement of the Rome III Committees has proposed standardized definitions for FD [7–10], there remains controversy, particularly about the highly heterogeneous manifestations and overlap with other GI disorders [11, 12]. So far, empirical approaches are still employed for the treatment of FD, and the current pharmacological options are limited and mostly based on individual symptoms [8, 13–15]. Improvement of patients with FD with current drug therapy is left with many unmet clinical needs, and the adverse effects associated with drugs also present challenges for clinicians and researchers [16–18].

As a nonpharmacological intervention, acupuncture is increasingly used in the treatment of FD and has been reported to be effective in altering acid secretion, GI motility, and visceral pain in patients with FD [19–21]. Moreover, as a type of physiotherapy, acupuncture treatment can avert the long-term side effects and resistance of drugs [22]. According to the previous research of literature and questionnaire survey conducted by our group, acupuncture and moxibustion therapy is effective in treating FD and is rated the I grade in the disease menu of acupuncture and moxibustion [23–27].

To date, despite the steady accumulation of evidence on inspecting the therapeutic effect of acupuncture for FD over the past decades, the available reports are still contradictive. The previous systematic reviews mainly claimed an inconclusive evaluation of acupuncture for treating FD, and the methodological flaws of those reviews might further magnify both clinical and methodological biases. Therefore, we conducted this updated systematic review and meta-analysis to critically evaluate the effectiveness of acupuncture for the treatment of FD, in order to provide more robust evidence for decision makers.

## 2. Method

This systematic review and meta-analysis was conducted according to the guidelines set forth in the Cochrane Handbook for Systematic Reviews of Interventions [28]. Reporting was conducted in accordance with the PRISMA guidance [29] (see Appendix S1 in Supplementary Material available online at http://dx.doi.org/10.1155/2016/3862916).

### 2.1. Literature Search

A comprehensive search of the following literature databases was conducted from their inception dates to Dec 31, 2015: Cochrane Central Register of Controlled Trials Library (CENTRAL), PubMed, EMBASE, Medline, Chinese Biomedical Database (CBM), Chinese National Knowledge Infrastructure (CNKI), Chinese VIP information (VIP), Wanfang Data, and Wanfang Dissertation Database. The search strategy is available in Appendix S2. We also searched the reference lists of all relevant papers for further studies. No language restrictions were applied in the search strategy.

### 2.2. Inclusion and Exclusion Criteria

RCTs that applied acupuncture as the therapeutic intervention for treating FD were included. The inclusion criteria were as follows.

1. *Types of Studies*. Only RCTs were included in our research. Quasi-RCTs (including trials that simply claimed to be randomized without any description of the method of random sequence generation), cross-over trials, cluster-randomized trials, and other study designs were excluded.
2. *Types of Participants*. Studies involving participants older than 17 years of age who met Rome II or Rome III diagnostic criteria for FD were included without limitations related to gender or race. Trials that observed the prognosis of FD coexisting with other disorders (e.g., depression and insomnia) were excluded in our review.
3. *Types of Interventions*. Trials that compared the effectiveness of acupuncture therapy with sham acupuncture or active control procedures were included. Particularly, in view of the uninvestigated heterogeneity across the diverse acupuncture types (including traditional manual acupuncture, acupressure, laser acupuncture, auricular needle, catgut implantation at acupuncture points, point injection, and emerging approaches including electroacupuncture and transcutaneous electrical stimulation (TES)), we only included RCTs that involved manual acupuncture or electroacupuncture as the therapeutic intervention for treating FD. As to the control interventions, RCTs using Chinese herbal medicine were excluded because of the ambiguous pharmacological mechanism and curative effect. Moreover, trials that only compared different forms of acupuncture were also excluded since we did not intend to investigate whether one type of acupuncture was more effective than another.
4. *Types of Outcome Measures*. Authoritative indicators [30] were concerned in our review for the assessment of symptoms and quality of life of FD patients, as follows: (1) primary outcomes: Nepean Dyspepsia Index (NDI) [31] (consisting of Nepean Dyspepsia Symptom Index (NDSI) and Nepean Dyspepsia Life Quality Index (NDLQI)); symptom scores (according to the National Guidelines for FD [32], Leeds Dyspepsia Questionnaire (LDQ) [33], gastrointestinal symptom score (GIS) [34], etc.); and the MOS 36-Item Short-Form Health Survey (SF-36) [35]; (2) secondary outcomes: ineffective rate and adverse events.

### 2.3. Article Selection

Two reviewers conducted the literature search independently (Cai for PubMed, Medline, EMBASE, Cochrane library, and Google Scholar as supplement; Hu for CBM, CNKI, VIP, Wanfang, and hand searching), and the third reviewer (Pang) organized the searched articles using the Endnote software.

After removing the duplicates, two reviewers (Jiang and Cai) independently evaluated the articles for relevance by title and abstracts. And full-texts of the remaining articles were obtained and screened out for eligibility by two reviewers (Cai and Hu). The third author (Pang) verified all the information and contacted the primary authors for unavailable articles if needed. Disagreements during article selection were resolved via discussion and consensus.

### 2.4. Data Extraction

Two reviewers (Jiang and Cai) extracted the information of each included trial into predefined data collection forms meeting the Cochrane standard. In the “characteristics of trails” form, studies were described in terms of author, country, participants, interventions, control types, frequency and treatment course, duration of one session, and main outcomes. If needed, primary authors of trials were contacted via email for providing the incomplete data. Furthermore, the “data extraction” form was used for recording and calculating the relevant data of the outcomes.

In some instances, the results of one study might be reported in several articles such as analyzing the results of one research in stages (in accordance with short term or long term of follow-up) or separately reporting the different outcomes of a study in different articles. For these instances, the multiple reports of the same research would be integrated together.

### 2.5. Quality Assessment

Two reviewers (Jiang and Li J) independently evaluated the risk of bias of the eligible studies using the Cochrane Collaboration's tool [36] and quantitatively estimated the quality of each included study according to the Modified Jadad Scale [37, 38]. Moreover, the Grading of Recommendations Assessment, Development, and Evaluation (GRADE system) was applied to confirm the quality of evidence and the strength of the recommendation [33].

According to the Cochrane risk of bias standards [39], each study was evaluated for its validity from the seven aspects: random sequence generation, allocation concealment, blinding of participants and personnel, blinding of outcome assessment, incomplete outcome data, selective reporting, and other bias. Similarly, included RCTs were scored according to the Modified Jadad Scale in five domains: Generation of allocation sequence (0–2 points), allocation concealment (0–2 points), blindness (0–2 points), and description of withdrawals and dropouts (0-1 points). Trials that scored 4–7 points were regarded to be of high quality, and those that scored 0–3 points were of low quality. What is more, according to the GRADE standards, four levels (high, moderate, low, and very low) were used for estimating the quality of evidence. Disagreements were resolved by consensus, and the third reviewer (Du) was consulted when disagreement persisted.

### 2.6. Statistical Analysis

Review Manager 5.2 software was utilized for statistical analysis on the basis of homogeneity of included trials. Risk ratios (RRs) and 95% confidence intervals (95% CIs) were applied for dichotomous data; and for continuous data, mean differences (MDs) and 95% CIs were used for analyses.

Statistical heterogeneity was assessed by chi-squared (*χ* ^2^ or Chi^2^) test and *I* ^2^ statistics [39]. According to the Cochrane standard, heterogeneity across the studies was regarded as substantial if Chi^2^ test resulted in a low *P* value (less than 0.10) and *I* ^2^ was greater than 50%. When contradiction was found (*P* < 0.01 while *I* ^2^ ≤ 50%; or *P* ≥ 0.01 while *I* ^2^ > 50%), *I* ^2^ statistic would be given priority for final interpretation of heterogeneity. A fixed-effect model was applied to calculate the pooled statistics in the absence of substantial heterogeneity (*P* ≥ 0.01 and/or *I* ^2^ > 50%). Conversely, if statistical heterogeneity was identified (*P* < 0.01, *I* ^2^ > 50%), causes of the heterogeneity would be detected first by subgroup analysis and/or sensitive analysis. If the heterogeneity could not readily be explained, a random-effects model would be applied. Results obtained by the random-effects model should be interpreted with caution.

## 3. Results

### 3.1. Description of Studies

#### 3.1.1. Literature Search

**Figure 1:**
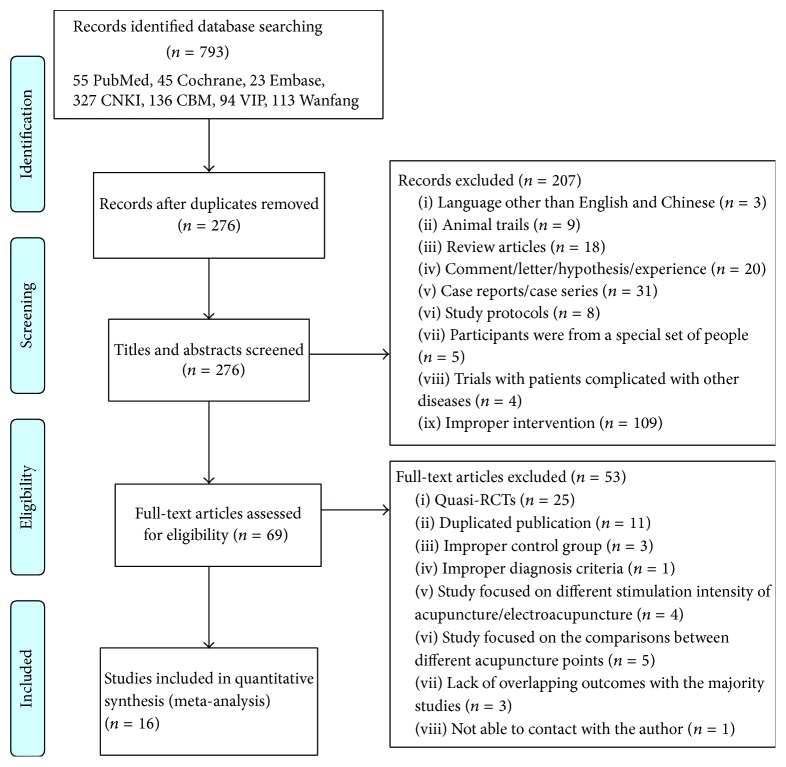
Systematic review process flowchart.

The electronic literature search yielded a total of 793 citations, of which 670 were published in Chinese and 123 in English. After removing the duplicates, 276 research articles remained for further consideration. A review of the titles and abstracts excluded 207 records based on relevance to the topic of interest, leaving 69 studies to be assessed for inclusion. Eventually, 16 articles were verified to meet our inclusion criteria [40–55]. Details can be seen in Figure 1.

#### 3.1.2. Study Characteristics

The characteristics of each trial were summarized in Table 1. All of the 16 articles were RCTs published from 2006 to 2015 and performed mainly in China (15) [40–44, 47–55] and South Korea (1) [46]. Two of the Chinese studies have been published in English journals registered by the Science Citation Index (SCI).

*(1) Participants*. The 16 RCTs recruited a total of 1436 individuals with FD (including 558 male and 878 female), with the sample sizes ranging from 48 to 354. Participants were enrolled as outpatients (or along with inpatients) in hospital settings. All trials limited the age range and medication use of participants; besides, the exclusion of structural abnormalities was identified in all of the included trials, among which three studies demanded previous gastroscopy of the individuals. 

*(2) Interventions/Controls*. Interventions involved manual acupuncture (in eight trials [40, 42, 44, 46–48, 50, 51]) or electroacupuncture (in eight trials [41, 43, 45, 49, 52–55]) alone. The most frequently used acupuncture points were ST36 (15 trials), PC6 (eight trials), CV12 (eight trials), ST25 (six trials), and LR3 (six trials). All but five trials [43, 44, 46, 50, 51] required the appearance of poststimulating “de-qi” sensation, a critical factor for the effectiveness of acupuncture therapy. As to control groups, sham acupuncture alone was adopted in six trials [42, 43, 45, 46, 49, 50], and nine trials [40, 41, 44, 47, 48, 51, 52, 54, 55] used medication as controls; one trial [53] applied both sham acupuncture and medication in separate control groups. As to the implementation of sham acupuncture, nonacupuncture points were used in all of the relevant trials, except one [42] that chose the sham points in different innervation area from the acupuncture group. Besides, prokinetic agents (including Itopride, Mosapride, and Domperidone) were used as controls among the included trials. 

*(3) Outcome Measures*. NDI was applied as the primary outcome in seven trials, among which, four trials [43, 45, 46, 50] reported both NDSI and NDLQI, and three trials [41, 51, 53] only focused on NDLQI. SF-36 was measured by six trials [40–42, 45, 49, 54]. Eight trials [40, 42, 47–49, 52–54] evaluated symptom scores of the participants, but with different scoring standards. Additionally, 11 [40, 41, 44, 45, 47, 48, 50–53, 55] trials estimated the effective/ineffective rate in accordance with various criteria. Safety assessment was reported in six trails.

### 3.2. Methodological Evaluation

**Figure 2:**
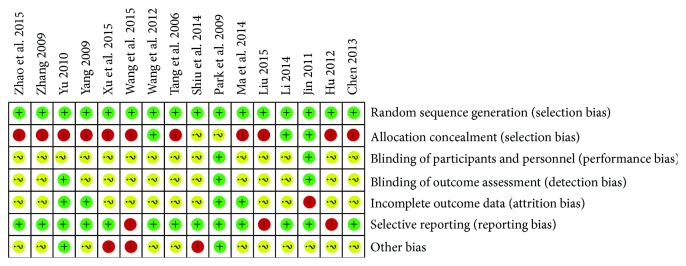
Risk of bias assessment.

**Figure 3:**
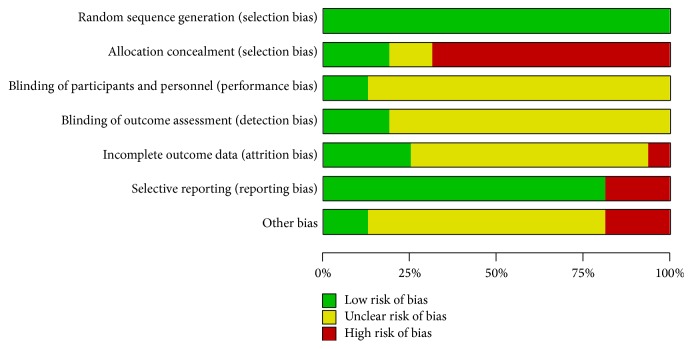
Risk of bias summary.

Results of the risk of bias assessment of the 16 RCTs are presented in Table S2 and Figure 2 and in summary in Figure 3. All trials ensured a defined method of randomization; however allocation concealment was considered inadequate in most of the studies. Another frequent bias was noted in blinding. For characteristics of acupuncture manipulations, it is hardly possible to conduct blinding, especially for the therapists and participants. Among the included RCTs, only two trials [42, 46] reported the blinding of participants, and three trials [42, 46, 53] blinded the statistical analysts. What is more, the majority of the included trials reported predefined or expected outcomes and selective reporting was not evident, except for three trials [41, 44, 50], in which the data of follow-up was omitted. By contrast, presentation of “incomplete outcome data” lacked in most of the trials, with only seven trials [41, 42, 45, 46, 52–54] reporting the details of dropouts.

In addition, results of assessment according to the Modified Jadad Scale [37] were presented in Table S1. Overall, Jadad scores of the included RCTs ranged from two to six points, with five of the trials rated as high quality, and 11 trials were regarded to be of low quality. What is more, in accordance with the GRADE system, most evidence from outcomes was found to be of low quality. Results were shown in Fig S1.

### 3.3. Data Analysis

#### 3.3.1. Evaluation on the Efficacy of Acupuncture Therapy

A comparison of acupuncture versus sham (placebo) acupuncture was first conducted in order to identify the curative effect of acupuncture therapy for FD. Overall, seven RCTs (including 636 participants) compared manual acupuncture [42, 46, 50] or electroacupuncture [43, 45, 49, 53] with sham acupuncture. 

**Figure 4:**
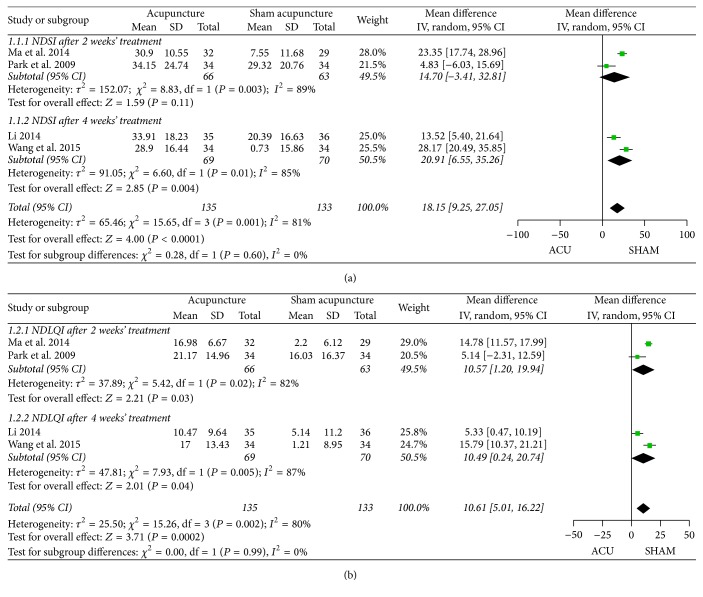
Forest plots of outcome “NDI.” Comparison: acupuncture versus sham acupuncture. (a) Comparative effect for NDSI. (b) Comparative effect for NDLQI.

*(1) NDI*. Five RCTs reported NDI scores as the main outcome (four trials [43, 45, 46, 50] reported both NDSI and NDLQI; one [53] estimated the four domains of NDLQI, resp.). Subgroup analysis of the first four trials was conducted according to the different duration of therapy (as shown in Figure 4). However, a high heterogeneity was shown for NDSI (2-week treatment: *P* = 0.003, *I* ^2^ = 89%; 4-week treatment: *P* = 0.01, *I* ^2^ = 85%) and NDLQI (2-week treatment: *P* = 0.02, *I* ^2^ = 82%; 4-week treatment: *P* = 0.005, *I* ^2^ = 87%). In view of the different symptom severity and the subjective inference of evaluating, we finally applied a random-effects model. The results demonstrated a favorable effect of acupuncture on NDSI after 4-week treatment (MD 20.91, 95% CI 6.55 to 35.26, *P* = 0.004) compared with sham acupuncture, while no difference was found after 2-week treatment (MD 14.70, 95% CI −3.41 to 32.81, *P* = 0.11). Correspondingly, the difference was distinct for NDLQI after both 2 (MD 10.57, 95% CI 1.20 to 19.94, *P* = 0.03) and 4 weeks (MD 10.49, 95% CI 0.24 to 20.74, *P* = 0.04) of treatment, while the pooled estimate of subgroups indicated the benefit of acupuncture in reducing NDSI (*P* < 0.0001) as well as improving NDLQI (*P* = 0.0002) in comparison with sham acupuncture. The one trial [53] that estimated the four domains (interference, know/control, eat/drink, and sleep/disturb) of NDLQI also yielded to a beneficial effect of acupuncture in improving the life quality of FD patients. Additionally, four [43, 45, 50, 53] trials reported the NDSI and NDLQI during the follow-up period (ranging from one to five months after the therapy), and significant difference was detected at each time-point of follow-up between intervention and control groups, which implied that acupuncture would improve NDI scores at follow-up period more than sham acupuncture would.

*(2) Symptom Scores*. All of the seven RCTs reported the symptom scores as a primary outcome. However, the reports were hardly homogeneous because of the biased methodology of scoring standards. Therefore, descriptive analysis was conducted for assessing the symptom scores across the trials.

Individually, one trial [42] reported the scores of the four major symptoms of FD (postprandial fullness, early satiation, epigastric pain, and epigastric burning), with each item scoring 0–4 points. Two trials [49, 53] evaluated the four major symptoms above with a scoring range of 0–3 points for each item; however, one trial [49] reported both dichotomous and continuous data and the other [53] estimated scores of the four symptoms separately. LDQ [33] was used to evaluate symptoms of FD patients in one trial [43]. And one trial [45] assessed the symptoms in reference to the clinics norms for FD formulated by Chinese Association of Integrative Medicine [32], with 12 symptoms being self-scored in four levels (0, 3, 5, and 7 points) by FD patients. Despite the biased methodology, the results of all the five RCTs revealed a comparative benefit of acupuncture for improving the symptoms of FD patients compared to sham acupuncture.

**Figure 5:**
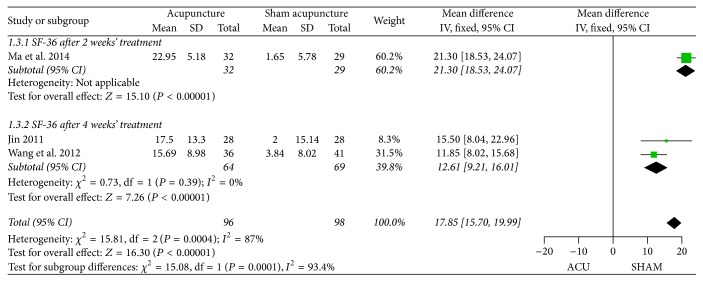
Forest plots of outcome “SF-36.” Comparison: acupuncture versus sham acupuncture.

*(3) SF-36*. Heterogeneity was detected (*P* = 0.00004, *I* ^2^ = 87%) across the three trials [42, 45, 49] that reported SF-36, which might be caused by different length of therapy as well as the bias on the self-rating of symptoms among the participants. Subgroup analysis was performed in accordance with distinct duration of treatment (Figure 5). The results demonstrated a favorable effect of acupuncture in enhancing the life quality of FD patients compared to sham acupuncture (2-week treatment: MD 21.30, 95% CI 18.53 to 24.07, *P* < 0.00001; 4-week treatment: MD 12.61, 95% CI 9.21 to 16.01, *P* < 0.00001).

**Figure 6:**
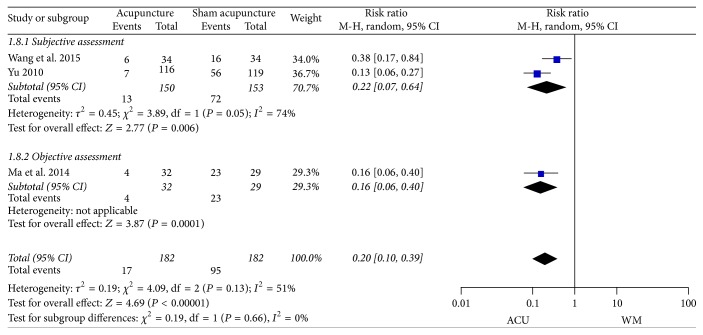
Forest plots of outcome “ineffective rate.” Comparison: acupuncture versus sham acupuncture.

*(4) Ineffective Rate*. The pooled results of the three trials [45, 50, 53] that reported clinical effective rate showed a moderate heterogeneity (*I* ^2^ = 51%), which might come from the different evaluating criteria. Therefore subgroup analysis was conducted according to the biased assessment methodology of effective rate, the subjective/objective approaches (Figure 6). The former was based on self-evaluation of patients or therapists, and the later mainly resorted to the grades of symptom scores. However, heterogeneity was still detected within subgroups, which was likely affected by the subjective differentiation during the evaluation. Given this, we eventually applied a random-effects model. Results of subgroup analysis verified the therapeutic effect of acupuncture versus sham acupuncture (subjective assessment: RR 0.22, 95% CI 0.07 to 0.64, *P* = 0.006; objective assessment: RR 0.16, 95% CI 0.06 to 0.40, *P* = 0.0001; total: RR 0.20, 95% CI 0.10 to 0.39, *P* < 0.00001). 

*(5) Adverse Effects*. Safety evaluation was conducted in two trials that compared the efficacy of acupuncture with sham acupuncture. The incidence rate of adverse effects in one trial [53] was 1.69% as reported, with five of the participants of sham acupuncture group experiencing subcutaneous hematoma or painful sensation during the process. Another trial [42] reported three participants that had felt electric shock or tingling during manipulation of needles. All the cases reported of adverse effects were recovered with reasonable countermeasures.

### 3.4. Evaluation on the Superiority of Acupuncture Therapy

In a further step, comparative analysis was conducted for the efficacy of acupuncture versus conventional medication, in order to explore the relative advantages of acupuncture therapy. Ten RCTs (containing 916 participants) were included. Overall, prokinetic agents (including Itopride, Mosapride, and Domperidone) were used by all the trials as controls. Despite the differential pharmacological mechanism of Domperidone versus the others, data of the three drugs were combined in view of their same therapeutic function of improving gastrointestinal motility.

**Figure 7:**
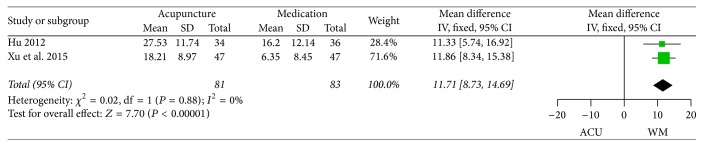
Forest plots of outcome “NDI” (NDLQI). Comparison: acupuncture versus medication.

*(1) NDI*. None of the ten RCTs reported NDSI, whereas three trials [41, 51, 53] conducted comparative analysis on NDLQI between acupuncture and medication groups. The pooled results of two trials [41, 51] implied a significant difference in favour of acupuncture, with no heterogeneity (Figure 7, MD 11.71, 95% CI 8.73 to 14.69, *P* < 0.00001). And the other trial [53] reported the respective scores of 4 domains (interference, know/control, eat/drink, and sleep/disturb) of NDLQI and yielded to a significant difference between intervention and control groups in the first three domains.

**Figure 8:**
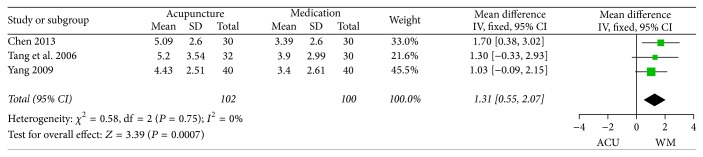
Forest plots of outcome “symptom scores.” Comparison: acupuncture versus medication.

*(2) Symptom Scores*. Nine RCTs reported symptom scores of FD patients; however the scoring criteria varied. To begin with, the four major symptoms of FD were scored (each item was scored 0–3 points) in five RCTs [40, 48, 52–54], among which, three trials [40, 48, 52] reported the total scores of the four symptoms and the other two [53, 54] calculated respectively for each of the symptoms. The pooled data of the former three trials showed a significant difference between acupuncture and medication groups in reducing the symptom scores of FD patients (MD 1.31, 95% CI 0.55 to 2.07, *P* = 0.0007, Figure 8).

**Figure 9:**
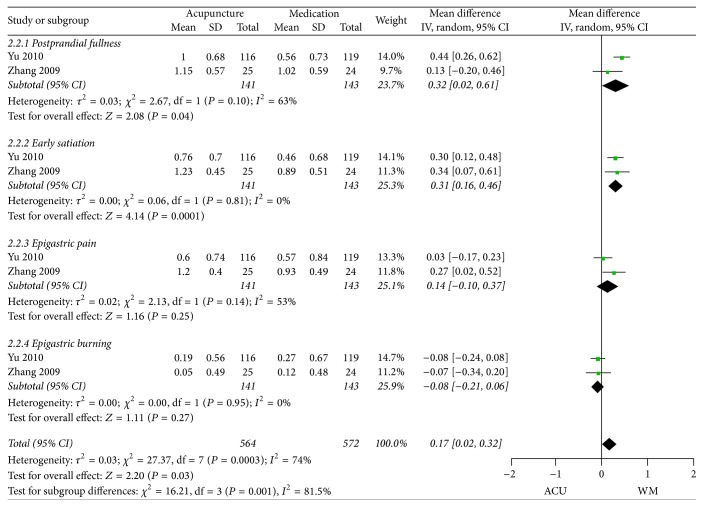
Forest plots of outcome “symptom scores” (individual scores of the four major symptoms). Comparison: acupuncture versus medication.

What is more, subgroup analysis was conducted for the other two trials based on the different symptoms. Moderate heterogeneity was detected within the two subgroups (*I* ^2^ = 63%; *I* ^2^ = 53%), and that was probably due to the subjective dissimilarity when scoring, which was hardly evitable. Therefore we adopted a random-effects model here (Figure 9). According to the subgroup analysis, acupuncture might be superior to medication in improving postprandial fullness (MD 0.32, 95% CI 0.02 to 0.61 *P* = 0.04) and early satiation (MD 0.31, 95% CI 0.16 to 0.46, *P* = 0.0001) of FD patients, whereas no difference was found in relieving epigastric pain (MD 0.14, 95% CI −0.10 to 0.37, *P* = 0.25) and epigastric burning (MD −0.08, 95% CI −0.21 to 0.06,  *P* = 0.27). However, the pooled effect size of the four subgroups revealed a beneficial effect of acupuncture in improving the major symptoms of FD patients compared to medication (MD 0.17, 95% CI 0.02 to 0.32, *P* = 0.03).

In addition, descriptive analysis was conducted for the other four RCTs [41, 44, 47, 51] that reported symptoms scores according to different standards. Two of the trials [41, 51] graded four ranks (0, 3, 5, and 7 points) of the symptoms, but the reference source of scoring criteria varied; one trial [44] separately scored the major and secondary symptoms with a range from 0 to 6 points; and the other trial [47] evaluated the five major symptoms. In spite of the methodological heterogeneity, all of the four RCTs affirmed the therapeutic effect of acupuncture for improving the symptoms of FD patients in comparison with medication.

**Figure 10:**
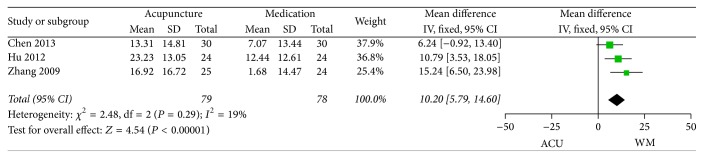
Forest plots of outcome “SF-36.” Comparison: acupuncture versus medication.

*(3) SF-36*. Three trials [40, 41, 54] reported SF-36 between acupuncture and medication groups after 4-week treatment, and the merged results (MD 15.24, 95% CI 5.79 to 14.60, *P* < 0.00001, Figure 10) implied a comparative superiority of acupuncture therapy in improving the quality of life of FD patients compared with medication, with a low heterogeneity (*P* < 0.00001, *I* ^2^ = 19%). 

**Figure 11:**
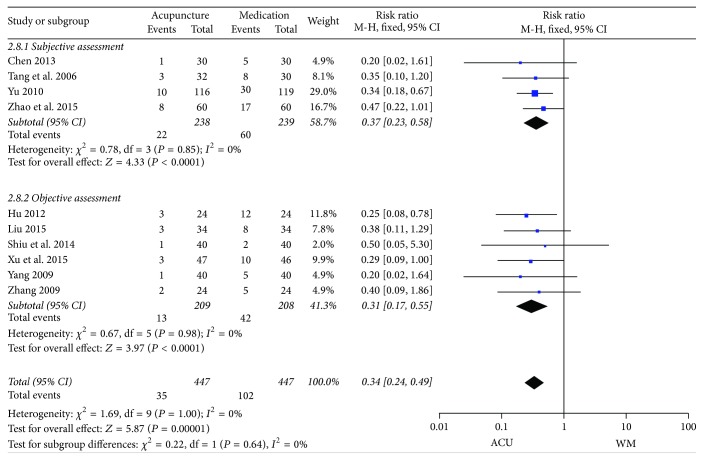
Forest plots of outcome “ineffective rate.” Comparison: acupuncture versus medication.

*(4) Ineffective Rate*. All of the ten RCTs reported effective rate after treatment between acupuncture and medication groups. Subgroup analysis was conducted due to the methodological heterogeneity across the trials (Figure 11). The pooled data demonstrated a more favorable effect of acupuncture therapy than medication, with no heterogeneity (subjective assessment: RR 0.37, 95% CI 0.23 to 0.58, *P* < 0.0001, *I* ^2^ = 0%; objective assessment: RR 0.31, 95% CI 0.17 to 0.55, *P* < 0.0001, *I* ^2^ = 0%).

*(5) Adverse Effects*. One trial [53] reported side effect (dizziness and headache) in one patient of medication group during the treatment, and the case recovered completely after drug withdrawal. No adverse effects data were reported in other studies examining acupuncture versus medication.

## 4. Discussion

### 4.1. Summary of Evidence

Irregular hierarchy of evidence quality always undermines the persuasion of synthesized results [57], which impels us to conscientiously formulate the methodology of our systematic review. Here, via rigorously screened 16 RCTs, we presented an unambiguous evaluation on the therapeutic effect of acupuncture for FD. Even so, however, caution is still warranted when considering the generalizability of our results due to the inevitable bias and quality issues of the evidence.

The outcome assessment of our systematic review was summarized in two aspects: symptoms (displayed in NDSI and symptom scores) and quality of life (reflected in NDLQI and SF-36) of FD patients. To begin with, the results of our meta-analysis revealed significant differences in acupuncture versus sham acupuncture for lowing NDSI and symptom scores after four-week treatment, while no significance was detected for NDSI after two weeks' therapy. What is more, compared with medication, acupuncture had beneficial effect in reducing the symptom scores of FD patients after four-week treatment, which was also supported by our meta-analysis. Particularly, superiority of acupuncture versus medication was detected in improving postprandial fullness and early satiation of FD patients; in contrast, the evidence was insufficient for demonstrating the comparative benefit of acupuncture in reliving epigastric pain and burning.

On the other hand, according to our review, acupuncture had superior effect in improving NDLQI and SF-36 compared to sham acupuncture in both 2-week and 4-week detection. Similarly, acupuncture was verified more effective in improving the life quality of FD patients than medication—an obvious improvement of NDLQI and SF-36 scores of patients was shown after the end of treatment.

Moreover, regarding ineffective rate, our meta-analysis implied that acupuncture was superior to either sham acupuncture or medication. Safety evaluation was evaluated in merely three trials, which should be further supplemented with relevant researches.

### 4.2. Strength

Compared with the previous published systematic reviews [58–62] on FD, vigorous quality assessment was conducted in our review against the flaws of the past articles. Comparative characteristics of each review were summarized in Table S2. Quasi-RCTs were included in previous reviews, of which the random sequence generation was either incorrect or with totally no description. Besides, no restriction had been imposed on interventions in some reviews [58–61]. Despite the comprehensive collection and the large number of individuals on which those estimate was based, different mechanism and curative properties of acupuncture therapies might give rise to an equivocal conclusion. Correspondingly, strength of the evidence might relatively decline due to the highly heterogeneous combination of interventions, and the synthesized results would provide vague information for the decision makers. In addition, as to the outcome measures, most of the reviews above were short of the evaluation on quantitative data from scales, especially NDI, an acknowledged scale that is specifically adopted for the assessment of FD [30].

Most of the previous systematic reviews confirmed the superiority of acupuncture for FD in comparison with sham acupuncture/medication, except two articles [58, 62] that reported no significant difference between acupuncture and medication groups after treatment. However, heterogeneous inclusion criteria and low quality of evidence of those reviews might cripple the robustness of evidence. Compared with the previous work, vigorous quality assessment was conducted in our review, for the sake of a more convincing estimate on acupuncture therapy for FD.

### 4.3. Limitations

Despite rigorous criteria of inclusion and methodology, limitations of our systematic review should be taken seriously into account.

To begin with, due to the barrier of language, we just screened RCTs published in English and Chinese, which may cause an omission of the eligible trials, especially those performed in other Asian regions where acupuncture therapy gains prevalence. Moreover, in spite of rigorous methodology, upon which the synthetic evidence was estimated and stratified, subjective views were inserted during the quality assessment of evidence. Another factor that may degrade the available evidence lies in the extensive inconsistency across the studies. Correspondingly, the random-effects model of meta-analysis was used to incorporate the heterogeneity among trials, but with more weight being awarded to smaller studies than such studies might receive in a fixed-effect meta-analysis, thus a wider confidence interval around the pooled estimate [39]. Consequently, all current evidence might not be sufficiently robust against potential methodological flaws and significant heterogeneity.

What is more, as mentioned above, confidence in recommendations may decrease if studies have major limitations that may bias their estimates of the treatment effect [63]. First of all, the participants of the 16 included RCTs aged 18 to 68 years, and the range of the courses of FD varied from three months to 40 years. Such extensive biases of demography may lead to a lack of universality of the synthesized data. Secondly, physicians' inconsistent discrimination of FD and self-reporting episodes among the patients may also bias the estimate toward or away from the null. Thirdly, highly heterogeneous application of acupuncture is probably a bigger impediment to the consistency between studies. Different from clinical drug trials, acupuncture therapies bias extensively along with acupuncture point selection/location, manipulation frequency, duration of stimuli, parameters of electroacupuncture, physicians' background, and so forth. Particularly, it has been verified that different manipulations and electrical parameters may exert different therapeutic effects [64, 65]. Moreover, acupuncture therapy is hardly possible to be “blinded.” On one hand, subjective factor of therapists may bias their manipulation between groups; on the other hand, acquaintance of acupuncture theory is also a great obstacle for blinding on participants, which will increase the withdrawal or dropout rate.

A further limitation comes with the outcomes across the studies. For one, recommended outcomes adhering to the updating diagnostic criteria of FD (e.g., NDI [31], LDQ [66], SF-36 [35] [30, 31, 67]) were seldom reported by the included RCTs. Besides, widespread use of the various scales (including valuators' appraisement and self-reporting of patients) is worth noticing, as the results may be biased due to subjective perception of physicians or patients [68, 69]. In addition, “efficacy rate” was reported widely among trials, but the referential significance may be devalued by its qualitative bias as well as the ambiguous grading standards of efficacy; and the necessity of other outcome measures reported among the included RCTs (including gastrointestinal hormone, Self-Rating Anxiety Scale (SAS), Self-Rating Depression Scale (SDS), Hamilton Anxiety Scale (HAMA), and gastric electrical parameters) is still disputable [67, 70].

### 4.4. Implications for Future Trials

Rigorously designed RCTs are expected referring to the most advanced and authoritative evaluation criteria, such as CONSORT (Consolidated Standards of Reporting Trials) statement and STRICTA (standards for reporting interventions in clinical trials of acupuncture) [66, 71–73]. Study protocols and designs should be performed strictly in case of any artificial bias during the process of clinical trials. First of all, the randomization should be rigorous and concealed to the greatest extent. Moreover, despite difficulties during the implementation of blinding for acupuncture therapists, blinding of patients, outcome assessors, and other care providers should be attempted in order to minimize the performance and detection biases. However, even though blinding is impossible for physicians and patients sometimes (especially in placebo-controlled trials), such studies can take other measures to reduce the risk of bias, such as treating patients according to a strict protocol to reduce the risk of differential behaviors by patients and physicians [36]. Here, we try to bring forward an assumption of whether participation of outsiders can be feasible for reducing the “performance bias” of the researchers, that is, to perform the trials through the provisional involvement of professional acupuncture manipulators who hold no interest relations with relevant studies.

Since FD is a chronic disease that is well known to wax and wane, a longer follow-up period with serial measurements of outcomes is also suggested to determine the validity of acupuncture in the long-term courses. And the particular subgroups of FD should be focused on in future researches, including different types of patients (in the light of various background, ages, courses, severity, etc.), subtypes of FD, and differentiating syndromes according to the theory of Traditional Chinese Medicine.

What is more, respective features of various types of acupuncture therapy should not be confounded, and the dose-effect relationship of acupuncture manipulation is also worthy of further clarification [74–77]. Moreover, the essential element of acupuncture effect, “de-qi” sensation, and its relative influence factors (e.g., intensity and duration of stimuli, physical condition, tolerance, and psychological factors) are expected to have more attention [78–82]. It is worth noting that although electroacupuncture has been regarded as a potent alternative of manual acupuncture [83], whether it works equivalently to the traditional one is still controversial [84, 85]. Furthermore, the optimum selection of acupuncture points is expected for further research to treat FD. As reported in the “result section,” except for ST36, PC6, CV12, ST25, and LR3, other acupuncture points varied extensively among the included RCTs, which might give rise to the subsequent ambiguity of pooled effect of acupuncture treatment.

Development of disputable placebo controls for acupuncture studies is also worth exploring in the future researches, in case of any misinterpretation on the results of clinical trials [86]. Numerous assumptions of sham acupuncture have been published over the years, but the highlights were always on the new-tech instruments, different intensity or depth of stimuli, and the control of patients' reaction of “de-qi” [86–90]. According to the theory of Traditional Chinese Medicine, the whole system of meridians and collaterals spreads all around the body, and acupuncture points cannot be limited to the certain points of the body [91, 92]. Therefore despite all kinds of attempts, as long as the stimulating points are within the effective range of the conventional acupuncture points, corresponding effect will raise. Therefore, more focus is expected on the meridians and acupuncture points that may be specific for the diseases being studied and the best prescriptions of acupuncture points for treating certain diseases.

## 5. Conclusion

In conclusion, on the basis of current clinical evidence, this systematic review suggests that acupuncture therapy (including manual and electroacupuncture) achieved statistically significant effect in improving the overall symptoms and quality of life of FD patients comparing to sham acupuncture and is superior to medication (prokinetic agents) in ameliorating the major symptoms of FD (postprandial fullness and early satiation). However, the current evidence may not be sufficiently robust against potential methodological flaws and significant heterogeneity. Further large-scale, well-designed RCTs on this topic are still warranted.

## Supplementary Material

Appendix S1. Search strategies.
S1 Fig. GRADE of the included trails.
S1 Table. Quality assessment of the included trials.
S2 Table. Comparisons between systematic reviews.

## Figures and Tables

**Table 1:** Characteristics of included studies

Study	Diagnosis criteria	Sample size (I/C)	Characteristics of participants (genders, ages, courses of disease); I/C		Intervention group^*∗*^ (main acupuncture points)	Frequency and treatment course	Control group^*∗*^	Duration of one session	Main outcomes
Chen 2013 [40]	Rome III	30/30	12M/18F 45.3 ± 11.8 20.5 ± 7.8	14M/16F 46.2 ± 12.3 20.6 ± 7.6	MA (CV12; ST36, PC6, LR3 of both sides; CV17)	Once daily, 5/w for 4 w	Itopride hydrochloride tablets (50 mg taken orally three times a day, 30 min before each meal)	30 min	Symptom scores; SF-36; ineffective rate

Hu 2012 [41]	Rome III	34/36	12M/22F 45.21 ± 9.37 23.68 ± 14.65	16M/20F 44.81 ± 8.95 23.89 ± 13.13	EA (CV12; ST25, PC6, ST36 of both sides)	Once daily, 5/w for 4 w	Itopride hydrochloride tablets (50 mg taken orally three times a day, 30 min before each meal)	30 min	NDI (NDLQI); symptom scores; SF-36; ineffective rate

Jin 2011 [42]	Rome III	28/28	11M/17F 49.29 ± 10.32 12.2 ± 12.2 y	10M/18F 48.25 ± 11.4 12.11 ± 10.20	MA (ST36, KI3 of both sides)	Every other day, 3~4/w for 4 w	Sham MA (nonacupoints, different area of innervation with the main acupoints of MA group)	No retention. If no “de-qi,” retain for 20–60 min	Symptom scores; SF-36

Li 2014 [43]	Rome III	35/36	12M/23F 37.78 ± 12.83 6.35 ± 8.09 y	13M/23F 34.47 ± 14.2 8.55 ± 9.59	EA (ST36, PC6 of one side; switch the sides each time)	Once daily, 5/w for 4 w	Sham EA (nonacupoints, three points in upper limbs and one near ST36)	30 min	NDI (NDSI, NDLQI)

Liu 2015 [44]	Rome III	34/34	19M/15F 38 ± 9 15.54 ± 6.77	18M/16F 38 ± 9 14.82 ± 7.31	MA (CV12; PC6, LR14, ST25, ST36, LR3, LR2 of both sides)	Once daily, 7/w for 2 w	Domperidone (10 mg taken orally three times a day, 30 min before each meal)	30 min	Symptom scores; ineffective rate

Ma et al. 2014 [45]	Rome III	32/29	17M/15F 36 ± 5 45 ± 7.1 m	13M/16F 34 ± 5 46 ± 8.8	EA (CV12; ST25, ST36 of both sides)	Once daily, 6/w for 2 w	Sham EA^*∗*^ (nonacupoints, near the main acupoints of EA group)	30 min	NDI (NDSI, NDLQI); symptom scores; SF-36; ineffective rate

Park et al. 2009 [46]	Rome II	34/34	8M/26F 30.71 ± 8.54	6M/28F 34.12 ± 10.36	MA (LI4, LR3, ST36, PC6, SP4 of both sides; CV12)	Once daily, 3/w for 2 w	Sham EA (nonacupoints, 1 cm away from the classical acupoints of MA group)	15 min	NDI (NDSI, NDLQI)

Shiu et al. 2014 [47]	Rome III	40/40	22M/18F 42 6 m–10.5 y	19M/21F 41 7.5 m–12 y	MA (SP6, SP9, SJ6 of both sides)	Once daily, 7/w for 3 w	Mosapride citrate dispersible tablets (5 mg taken orally three times a day)	30 min	Symptom scores; ineffective rate

Tang et al. 2006 [48]	Rome II	32/30	15M/17F 38.2 ± 11.3 3 m–10 y	14M/16F 36.7 ± 12.8 3 m–9 y	MA (ST36, ST44, LR3, PC6, BL20, BL21, BL18 of both sides; CV12)	Once daily for 3 courses; 10 d/course	Domperidone (10 mg taken orally three times a day, 30 min before each meal)	30 min	Symptom scores; ineffective rate

Wang et al. 2015 [49]	Rome III	36/41	14M/22F 33.1 ± 1.2 66.58 ± 5.16	12M/29F 32.6 ± 1.2 47.29 ± 4.04	EA (ST42, ST40, ST36, ST34 of both sides)	Once daily, 5/w for 4 w	Sham EA (nonacupoints, three in upper limbs and one near ST36)	30 min	Symptom scores; SF-36

Wang et al. 2012 [50]	Rome III	34/34	10M/24F 41.8 years old 5.48 years	8M/26F 44.6 years old 8.29 years	MA (ST36, PC6 of both sides)	Once daily, 5/w for 4 w	Sham EA (nonacupoints, near the main acupoints of MA group)	30 min	NDI (NDSI, NDLQI); ineffective rate

Xu et al. 2015 [51]	National Guideline^*∗*^	47/46	21M/25F 39.74 ± 10.28 2.01 ± 0.19	20M/27F 38.76 ± 9.52 2.42 ± 0.53	MA (PC6, ST36, ST25 of both sides; CV6, CV10, CV12, CV13, EX-HN3, DU24, DU20)	Once daily, 5/w for 4 w	Domperidone (10 mg taken orally three times a day, 15 min before each meal)	30 min	NDI (NDLQI); symptom scores; ineffective rate

Yang 2009 [52]	Rome III	40/40	18M/22F 46.2 ± 11.7 14.5 ± 7.8 m	21M/19F 45.9 ± 12.1 14.7 ± 7.6	EA (ST42, ST40, ST36, ST34 of both sides)	Once daily, 5/w for 4 w	Itopride hydrochloride tablets (50 mg taken orally three times a day, 30 min before each meal)	30 min	Symptom scores; ineffective rate

Yu 2010 [53]	Rome III	116/119/119	33M/82F 38.68 ± 13.51 48 m	34M/83F 37.18 ± 13.03 37 m 36M/81F 37.32 ± 13.79 47 m	EA (ST42, ST40, ST36, ST34 of both sides)	Once daily, 5/w for 4 w	Sham EA (nonacupoints, three in upper limbs and one near ST36) Itopride hydrochloride tablets (50 mg taken orally three times a day, 30 min before each meal)	30 min	NDI; symptom scores; ineffective rate

Zhang 2009 [54]	Rome III	24/24	13M/11F 35.7 ± 10.43 18.4 ± 12.72	14M/10F 35.23 ± 11.25 18.17 ± 13.54	EA (CV12; BL21 of both sides; ST36 of one side)	Once daily, 5/w for 4 w	Itopride hydrochloride tablets (50 mg taken orally three times a day, 30 min before each meal)	30 min	Symptom scores; SF-36; ineffective rate

Zhao et al. 2015 [55]	Rome III	60/60	28M/32F 40.22 ± 10.18 8.6 ± 3.8 y	25M/35F 38.5 ± 9.1 7.4 ± 2.2 y	EA (LR3, ST36, ST25, BL21, SP9 of both sides at specific time according to “midnight-noon ebb-flow” theory)	Once daily, 3/w for 2 w	Itopride hydrochloride tablets (5 mg taken orally three times a day)	30 min	Ineffective rate

MA, manual acupuncture; EA, electroacupuncture; NDI, Nepean Dyspepsia Index; NDSI, Nepean Dyspepsia Symptom Index; NDLQI, Nepean Dyspepsia Life Quality Index; SF-36, the MOS 36-Item Short-Form Health Survey.

^*∗*^National Guideline: Guidelines for the diagnosis and treatment of dyspepsia in China (Dalian, 2007) [56].
