# Supplementary material for: Acupuncture for Functional Dyspepsia: What Strength Does It Have? A Systematic Review and Meta-Analysis of Randomized Controlled Trials
Source: Evid Based Complement Alternat Med. 2016 Dec 29;2016:3862916. doi: 10.1155/2016/3862916 (PMC5227170; doi:10.1155/2016/3862916)
Supplement: Supplementary file 1 — Appendix S1. Search strategies. S1 Fig. GRADE of the included trails. S1 Table. Quality assessment of the included trials. S2 Table. Comparisons between systematic reviews. [file 3862916.f1.pdf]

S1 Appendix. Search strategy

1. PUBMED

Search terms ： (("acupuncture"[MeSH Terms] OR "acupuncture"[All Fields] OR "acupuncture therapy"[MeSH Terms] OR ("acupuncture"[All Fields] AND "therapy"[All Fields]) OR "acupuncture therapy"[All Fields]) OR "acupuncture therapy"[MeSH Terms]OR ("acupuncture"[All Fields] AND "treatment"[All Fields]) OR "acupuncture treatment"[All Fields]) OR ("electroacupuncture"[MeSH Terms] OR "electroacupuncture"[All Fields])) AND ("dyspepsia"[MeSH Terms] OR "dyspepsia"[All Fields] OR "functional dyspepsia"[MeSH Terms] OR "functional dyspepsia"[All Fields] OR "indigestion"[All Fields] OR "indigestions"[All Fields] OR "distention"[All Fields] OR "non-ulcer dyspepsia"[All Fields])

2. EMBASE

Search terms ： ('acupuncture'/exp OR acupuncture OR 'electroacupuncture'/exp OR electroacupuncture OR 'acupuncture therapy'/exp OR acupuncture therapy OR 'acupuncture treatment'/exp OR acupuncture treatment) AND ('dyspepsia'/exp OR dyspepsia OR 'functional dyspepsia'/exp OR functional dyspepsia OR indigestion OR indigestions OR distention OR non-ulcer dyspepsia)

3. Cochrane Central Register of controlled trials

Search terms ： (acupuncture OR electroacupuncture) AND (dyspepsia OR functional dyspepsia OR indigestion OR distention)

4. China National Knowledge Infrastructure (CNKI)

Search terms:(针刺 OR 电针 OR 针灸 OR 针灸疗法 OR 针刺疗法) AND (消化不良 OR 功能性消化不良 OR 积滞 OR 痞满 OR 胃脘痛 OR 非溃疡性消化不良)

5. Chinese Biomedical Database (CBM)

Search terms:(针刺 OR 电针 OR 针灸 OR 针灸疗法 OR 针刺疗法) AND (消化不良 OR 功能性消化不良 OR 积滞 OR 痞满 OR 胃脘痛 OR 非溃疡性消化不良)

6. Chinese VIP information (VIP)

Search terms:(针刺 OR 电针 OR 针灸 OR 针灸疗法 OR 针刺疗法) AND (消化不良 OR 功能性消化不良 OR 积滞 OR 痞满 OR 胃脘痛 OR 非溃疡性消化不良)

7. Wanfang Data and Wanfang Dissertation Database

Search terms:(针刺 OR 电针 OR 针灸 OR 针灸疗法 OR 针刺疗法) AND (消化不良 OR 功能性消化不良 OR 积滞 OR 痞满 OR 胃脘痛 OR 非溃疡性消化不良)

S1 Fig. GRADE

Summary of findings:

Acupuncture compared to Sham acupuncture for functional dyspepsia

Patient or population: functional dyspepsia
Setting: hospital inpatients and outpatients
Intervention: Acupuncture
Comparison: Sham acupuncture

Outcomes	Anticipated absolute effects* (95% CI)		Relative effect (95% CI)	№ of participants (studies)	Quality of the evidence (GRADE)	Comments
	Risk with Sham acupuncture	Risk with Acupuncture				
Nepean Dyspepsia Symptom Index after 2-week treatment (NDSI)		The mean nepean Dyspepsia Symptom Index after 2-week treatment in the intervention group was 14.7 more (3.41 fewer to 32.81 more)	-	129 (2 RCTs)	⊕⊕◯◯ LOW [1,2]	
Nepean Dyspepsia Symptom Index after 4-week treatment (NDSI) follow up: range 1 months to 5 months		The mean nepean Dyspepsia Symptom Index after 4-week treatment in the intervention group was 20.91 more (6.55 more to 35.26 more)	-	139 (2 RCTs)	⊕⊕◯◯ LOW [1,3]	
Nepean Dyspepsia Life Quality Index after 2-week treatment (NDLQI)		The mean nepean Dyspepsia Life Quality Index after 2-week treatment in the intervention group was 10.57 more (1.2 more to 19.94 more)	-	129 (2 RCTs)	⊕⊕◯◯ LOW [1,2]	
Nepean Dyspepsia Life Quality Index after 4 weeks' treatment (NDLQI) follow up: range 1 months to 5 months		The mean nepean Dyspepsia Life Quality Index after 4 weeks' treatment in the intervention group was 10.49 more (0.24 more to 20.74 more)	-	139 (2 RCTs)	⊕⊕◯◯ LOW [1,3]	
SF-36 after 2 week's treatment (SF-36 scale) follow up: mean 1 months	The mean SF-36 after 2 week's treatment was **1.65**	The mean SF-36 after 2 week's treatment in the intervention group was 21.3 more (18.53 more to 24.07 more)	-	61 (1 RCT)	⊕⊕◯◯ LOW [1]	

SF-36 after 4-week treatment (SF-36 scale) follow up: range 1 months to 3 months	The mean SF-36 after 4-week treatment in the intervention group was 12.61 more (9.21 more to 16.01 more)	-	133 (2 RCTs)	⊕⊕◯◯ LOW[1,4]

***The risk in the intervention group** (and its 95% confidence interval) is based on the assumed risk in the comparison group and the **relative effect** of the intervention (and its 95% CI).

CI: Confidence interval; **MD:** Mean difference

GRADE Working Group grades of evidence
High quality: We are very confident that the true effect lies close to that of the estimate of the effect
Moderate quality: We are moderately confident in the effect estimate: The true effect is likely to be close to the estimate of the effect, but there is a possibility that it is substantially different
Low quality: Our confidence in the effect estimate is limited: The true effect may be substantially different from the estimate of the effect
Very low quality: We have very little confidence in the effect estimate: The true effect is likely to be substantially different from the estimate of effect

1. High risk of allocation concealment and/or blinding approach
2. Inconsistent results between RCTs
3. Incomplete reporting data
4. Reasons of drop-outs largely relevant with intervention

Summary of findings:

Acupuncture compared to Medication for functional dyspepsia

Patient or population: functional dyspepsia
Setting: hospital inpatients and outpatients
Intervention: Acupuncture
Comparison: Medication

Outcomes	Anticipated absolute effects* (95% CI)		Relative effect (95% CI)	№ of participants (studies)	Quality of the evidence (GRADE)	Comments
	Risk with Medication	Risk with Acupuncture				
Nepean Dyspepsia Life Quality Index (NDLQI)		The mean nepean Dyspepsia Life Quality Index in the intervention group was 11.71 more (8.73 more to 14.69 more)	-	164 (2 RCTs)	⊕⊕◯◯ LOW [1,2]	
Symptom scores-total assessed with: total scores of four major symptoms with a range from 0-3 points respectively		The mean symptom scores-total in the intervention group was 1.31 more (0.55 more to 2.07 more)	-	202 (3 RCTs)	⊕⊕◯◯ LOW [1]	
Symptom scores-Postprandial fullness		The mean symptom scores-Postprandial fullness in the intervention group was 0.32 more (0.02 more to 0.61 more)	-	284 (2 RCTs)	⊕⊕◯◯ LOW [1]	
Symptom scores-Early satiation		The mean symptom scores-Early satiation in the intervention group was 0.31 more (0.16 more to 0.46 more)	-	284 (2 RCTs)	⊕⊕◯◯ LOW [1]	
Symptom scores-Epigastric pain		The mean symptom scores-Epigastric pain in the intervention group was 0.14 more (0.1 fewer to 0.37 more)	-	284 (2 RCTs)	⊕⊕◯◯ LOW [1,3]	
Symptom scores-Epigastric burning		The mean symptom scores-Epigastric burning in the intervention group was 0.08 fewer (0.21 fewer to 0.06 more)	-	284 (2 RCTs)	⊕⊕◯◯ LOW [1,3]	
SF-36		The mean SF-36 in the intervention group was 10.2 more (5.79 more to 14.6 more)	-	157 (3 RCTs)	⊕⊕◯◯ LOW [1,4]	

***The risk in the intervention group** (and its 95% confidence interval) is based on the assumed risk in the comparison group and the **relative effect** of the intervention (and its 95% CI).

CI: Confidence interval; **MD:** Mean difference

GRADE Working Group grades of evidence
High quality: We are very confident that the true effect lies close to that of the estimate of the effect
Moderate quality: We are moderately confident in the effect estimate: The true effect is likely to be close to the estimate of the effect, but there is a possibility that it is substantially different
Low quality: Our confidence in the effect estimate is limited: The true effect may be substantially different from the estimate of the effect
Very low quality: We have very little confidence in the effect estimate: The true effect is likely to be substantially different from the estimate of effect

1. High/Unclear risk of bias in allocation concealment and blinding
2. Inclusion criteria of trial was lack of authority
3. Inconsistent reporting results
4. Incomplete reporting data

S1 Table. Risk of bias assessment and Jadad scores

Study	Random Sequence Generation	Allocation Concealment	Blinding of Participants and Personnel	Blinding of Outcome Assessment	Incomplete Outcome Data	Selective Reporting	Other bias	Jadad Score
Chen 2013[40]	L*: Random number table	H*	U*	U	U	L	U	2
Hu 2012[41]	L: Random number table	H	U	U	U: 2/34 missing from acupuncture group without any probable reason reported	H: Incomplete data of follow-up	U	3
Jin 2011[42]	L: Random number table	L: Sequentially numbered, opaque, sealed envelopes	L: Patients were blinded	L: Outcome assessors and statistical analysts were blinded	H: 2/28 missing from acupuncture group (1 for "lack of efficacy"); 2/28 missing from sham-acupuncture group (both for "lack of efficacy"); reasons of missing was largely relevant with acupuncture method	L	U	6
Li 2014[43]	L: Central Randomization	L: Central Allocation	U	U	U	L	U	4
Liu 2015[44]	L: Random number table	H	U	U	U	H: Incomplete data of follow-up	U	2
Ma 2014[45]	L: Excel sheet	H	U	U	L: 3/35 missing from acupuncture group (one for fainting during acupuncture); 6/35 missing from sham-acupuncture group	L	U	3

S1 Table CONT.

Study	Random Sequence Generation	Allocation Concealment	Blinding of Participants and Personnel	Blinding of Outcome Assessment	Incomplete Outcome Data	Selective Reporting	Other bias	Jadad Score
Park 2009[46]	L: Blocked randomization	U	L: Patients were blinded	L:Investigators, study monitors were blinded	L: "All patients completed the study and there were no losses to follow up, no treatment withdrawals, no trial group changes and no major adverse events"	L	L：Protocol reported and authorized	4
Shui 2014[47]	L: Using a computer random generator	U	U	U	U	L	H: Incomplete statistical data of baseline evaluation; unclear source of methodology (symptom scores)	2
Tang 2006[48]	L: Random number table	H	U	U	U	L	U	2
Wang 2012[49]	L: Central Randomizatio n	L: Central Allocation (through text messages and e-mails)	U	U	U	L	U	4
Wang 2015[50]	L: Random number table	H	U	U	U	H: Incomplete data of follow-up	H: Unclear source of methodology (effective assessment)	2

S1 Table CONT.

Study	Random Sequence Generation	Allocation Concealment	Blinding of Participants and Personnel	Blinding of Outcome Assessment	Incomplete Outcome Data	Selective Reporting	Other bias	Jadad Score
Xu 2015[51]	L: Random number table	H	U	U	U	L	H: Inclusion Criteria was lack of authority	2
Yang 2009[52]	L: Random number table	H	U	U	L: "No dropout in this study "	L	U	3
Yu 2010[53]	L: Central Randomizatio n	L: Central Allocation	U	L: Outcome assessors and statistical analysts were blinded	L: 6/116 missing from acupuncture group; 4/119 missing from sham-acupuncture group; 4/119 missing from medicine group	L	L: Protocol reported and registered; specific quality control principle	6
Zhang 2009[54]	L: Random number table	H	U	U	U: 2 missing before the trails without any probable reason reported	L	U	3
Zhao 2015[55]	L: Random number table	H	U	U	U	L	U	2

*L, "low risk" of bias; U, "unclear risk" of bias; H, "high risk" of bias. According to Cochrane Collaboration's tool for assessing risk of bias, bias of each item can be judged as one of the three levels-Low risk, Unclear risk and High risk.

S2 Table. Comparisons across Related Systematic Reviews

Authors of Systematic Reviews	Inception Date	Types of Included Studies (numbers)	Numbers of Included studies	Countries of Included Trials	Quality Assessment	Intervention	Control	Reported Outcomes	Conclusions
Zhu [62]	2007.3	RCTs<br>Quasi-RCTs[1](8)	11	China	Simple approach method of Cochrane handbook[2]	MA<br>Moxibustion<br>PI<br>PCI<br>TENS	M<br>HM	Ineffective rate<br>Symptom scores<br>Adverse effect	AT>M[3]; PI>M; PCI<br>≈WM[3]; MB≈M;<br>ACU+HM≈HM
Wu [60]	Not mention	RCTs<br>Quasi-RCTs (5)	16	China	Jadad Scale	MA<br>EA<br>Moxibustion<br>MA+Moxibustion	Prokinetics	Total effective rate	Acupuncture and moxibustion therapy>WM
Leng [61]	2013.2.5	RCTs<br>Quasi-RCTs (8)	15	China	Cochrane risk of bias tool	MA<br>EA<br>MA+M[3]<br>EA+M<br>Finger AT	M	Total effective rate<br>Symptom scores<br>MTL<br>NDLQI<br>SF-36<br>Adverse effect	AT>WM<br>AT+WM>WM
Kim [58]	2013.8.20	RCTs<br>Quasi-RCTs (8)	20	China<br>Korea<br>Indonesia	Cochrane risk of bias tool	MA<br>Finger AT<br>MA+AA<br>MA+M	No treatment<br>Sham AT<br>M	Total effective rate<br>Symptom scores<br>(VAS NDI)<br>Adverse effect	AT (included Finger AT)>Sham AT<br>≈WM

S2 Table Cont.

Authors of Systematic Reviews	Inception Date	Types of Included Studies (numbers)	Numbers of Included studies	Countries of Included Trials	Quality Assessment	Intervention	Control	Reported Outcomes	Conclusions
Lan [59]	2012.2	RCTs<br>Quasi-RCTs (2)	6	China<br>Korea	Cochrane risk of bias tool<br>GRADE system	MA<br>EA<br>MA+M	Sham AT<br>M	Symptom scores<br>Frequency of symptoms<br>NDI<br>SF-36<br>SAS<br>SDS<br>Adverse effect	AT>Sham AT<br>AT≈WM
Pang	2015.12.31	RCTs	16	China<br>Korea	Cochrane risk of bias tool<br>GRADE system<br>Jadad scale	MA<br>EA	Sham AT<br>M	NDI (NDSI; NDLQI)<br>Symptom scores<br>SF-36<br>Ineffective rate<br>Adverse effect	AT>Sham AT<br>AT>WM

Abbreviation : AT, acupuncture treatment; MA, manual acupuncture; EA, electroacupuncture; PI, point injection; PCI, point catgut implantation; TENS, transcutaneous electrical nerve stimulation; AA, auricular acupoints; M, medication; HM, herbal medicine.

1.Quasi-RCTs: 1) RCTs with quasi-random method of allocating participants to different interventions, such as alternation, date of birth, or case record number. 2) RCTs with insufficient randomized method (only with a brief statement such as “we randomly allocated” or “using a randomized design”).

2.Descriptive assessment with respect to four aspects: random sequence generation, allocation concealment, blinding of participants and personnel, blinding of outcome assessment, withdrawal.

3.”+” indicated “combined”; “>” means the efficacy of former is superior to the later; “≈” means no significant difference exists on the efficacy between the former and the later.
